# Investigating the Heterogeneity of Immune Cells in Triple-Negative Breast Cancer at the Single-Cell Level before and after Paclitaxel Chemotherapy

**DOI:** 10.3390/ijms241814188

**Published:** 2023-09-16

**Authors:** Heng Zhao, Zhang Lin, Yangfan Zhang, Jingjing Liu, Qi Chen

**Affiliations:** 1 Fujian Key Laboratory of Innate Immune Biology, Biomedical Research Center of South China, Fujian Normal University Qishan Campus, College Town, Fuzhou 350117, China; zhao_heng@yeah.net (H.Z.); lin_zhang@fjnu.edu.cn (Z.L.); qbx20210160@yjs.fjnu.edu.cn (Y.Z.); liujingjing980618@163.com (J.L.); 2 College of Life Sciences, Fujian Normal University, Fuzhou 350117, China

**Keywords:** triple-negative breast cancer (TNBC), paclitaxel chemotherapy, single-cell sequence, T cells, ILCs

## Abstract

Despite the numerous treatments for triple-negative breast cancer (TNBC), chemotherapy is still one of the most effective methods. However, the impact of chemotherapy on immune cells is not yet clear. Therefore, this study aims to explore the different roles of immune cells and their relationship with treatment outcomes in the tumor and blood before and after paclitaxel therapy. We analyzed the single-cell sequencing data of immune cells in tumors and blood before and after paclitaxel treatment. We confirmed a high correlation between T cells, innate lymphoid cells (ILCs), and therapeutic efficacy. The differences in T cells were analyzed related to therapeutic outcomes before and after paclitaxel treatment. In the effective treatment group, post-treatment tumor-infiltrating CD8^+^ T cells were associated with elevated inflammation, cytokines, and Toll-like-receptor-related gene expression, which were expected to enhance anti-tumor capabilities in tumor immune cells. Moreover, we found that the expression of immune-checkpoint-related genes is also correlated with treatment outcomes. In addition, an ILC subgroup, b_ILC1-XCL1, in which the corresponding marker gene XCL1 was highly expressed, was mainly present in the effective treatment group and was also associated with higher patient survival rates. Overall, we found differences in gene expression in T cells across different groups and a correlation between the expression of immune checkpoint genes in T cells, the b_ILC1-XCL1 subgroup, and patient prognosis.

## 1. Introduction

Triple-negative breast cancer (TNBC) is characterized by a lack of expression of estrogen receptor (ER), progesterone receptor (PR), and human epidermal growth factor receptor 2 (HER2/neu) and exhibits high invasiveness, poor prognosis, and a high recurrence rate [1]. Current treatment methods for TNBC include surgery, chemotherapy, and radiation therapy [2]. Among these, chemotherapy remains the most effective treatment for TNBC [3] and is also one of the treatment methods for other tumors. Chemotherapy can induce immunogenic cell death, releasing a large number of damage-associated molecular patterns (DAMPs) and other signaling molecules and activating dendritic cells (DCs) to present tumor antigens to T cells, generating an anti-tumor immune response [4]. Chemotherapeutic drugs have a toxic effect on tumor cells, inhibiting their growth, but they can also affect normal cell growth and differentiation [5]. Therefore, the impact of chemotherapy on immune cells in the tumor microenvironment and non-tumor environment of patients has significant implications for tumor development.

Immune cells, including macrophages, T cells, B cells, and dendritic cells, within the tumor microenvironment participate in various aspects of TNBC progression [6] and play different roles during tumor development, having anti-tumor effects [7]. However, while the classic activation and polarization enhance anti-tumor effects [8], alternatively activated macrophage subgroups promote tumor progression [9]. Therefore, understanding changes in immune cells within the tumor microenvironment is crucial for tumor treatment. Particularly, tumors can have systemic effects on immunity [10]. They can cause the accumulation of immature neutrophils and monocytes, leading to immune suppression and influencing tumor progression through tumor infiltration [11,12]. They can also participate in the establishment of pre-metastatic niches in distant organs through homing mechanisms [13]. Current research on TNBC mainly focuses on the intrinsic immune characteristics of the tumor, and the relationship between changes in immune cells and tumor tissues is not yet clear.

**Figure 1:**
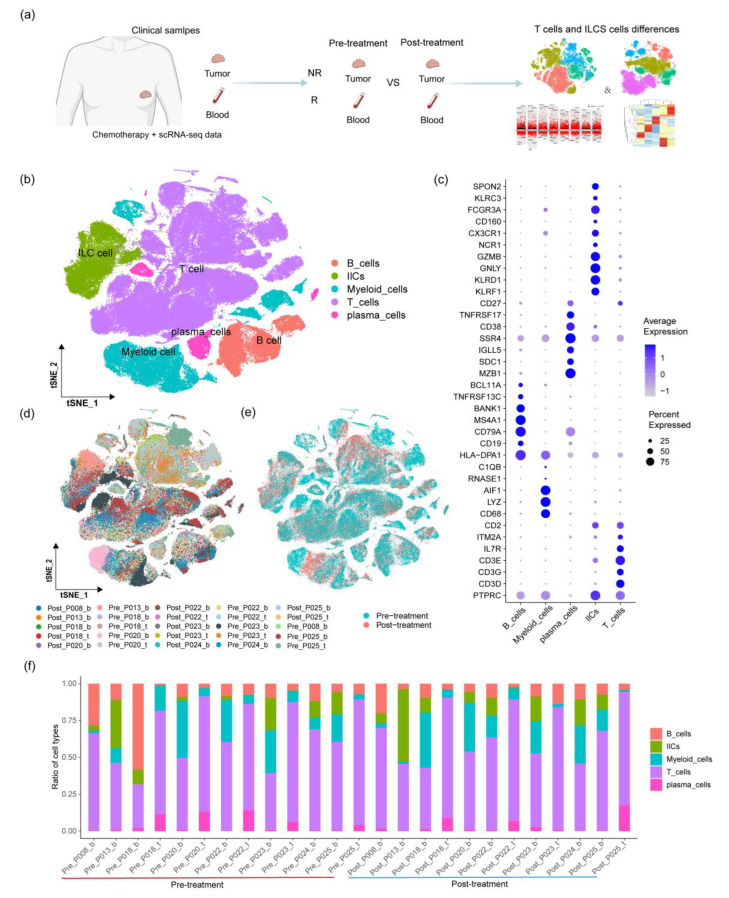
The landscape of the immune cell atlas for triple-negative breast cancer (TNBC). (**a**) The main process diagram of data analysis in this study. (**b**) The t-SNE plot was used to visualize the immune cell types and their distribution within TNBC. (**c**) The bubble plot was used to display the expression levels of major marker genes in different immune cells (consistent with the cell types labeled in (**b**)). (**d**,**e**) The t-SNE plot was used to display the immune cell types and their distribution in different samples before and after treatment. (**f**) The bar plot represents the proportion of immune cells in different samples.

In this study, we used single-cell data from TNBC treated with paclitaxel [14], including the data from tumors and immune cells in the blood and tumor tissues before and after treatment and clinical data from patients pre- and post-treatment. We explored the immune microenvironment of TNBC tumors and blood immune cells and reanalyzed the single-cell data according to the process outlined in Figure 1a. We found that T cells and ILCs are more relevant to changes in tumor biopsy lesions. We analyzed the subgroups of T cells and ILCs to compare the differences between patients across various tissues, before and after treatment, and under different treatment efficacy conditions. We investigated the changes in T cell genes in TNBC under different conditions during treatment and elaborated upon the relationship between ICG changes in T cells pre- and post-treatment and treatment efficacy, as well as the prognostic relevance of the b_ILC1-XCL1 subgroup.

## 2. Results

### 2.1. Constructing an Immune Cell Atlas for TNBC

Single-cell sequencing data of triple-negative breast cancer (TNBC) were obtained from the GEO database [14]. Initial screening of the raw data resulted in the selection of single-cell data treated exclusively with paclitaxel. The data were filtered based on factors such as the mitochondrial gene proportion, the red blood cell gene proportion, and the number of genes; dead cells, doublets, and red blood cells were excluded (Figure S1a–d). The obtained clean data consisted of 159,646 cells. Seurat was used for cell clustering and gene marker identification [15]. The t-SNE plot was used for dimensionality reduction and visualization, creating an immune cell landscape of TNBC.

The immune cell atlas included T cells, innate lymphoid cells (ILCs), plasma cells, B cells, and myeloid cells (Figure 1b). When clustering cells, plasma cells, which are terminally differentiated B cells with completely different transcription mechanisms, were distinguished from B cells [16]. The remaining subgroups were consistent with the cell types and distribution in the original data clustering (Figure S1f), demonstrating the accuracy of this reanalysis. These cells were identified as T cells based on gene markers, such as CD3D, CD3G, CD3E, and IL7R; as myeloid cells based on gene markers LYZ, C1QB, AIF1, and HLA-DPA1; as B cells based on gene markers CD19, CD79A, MSA4A1, and BANK1; as plasma cells based on gene markers IGLL1, MZB1, SDC1, IGLL5, and SSR4; and as ILCs based on gene markers KLRF1, KLRD1, GNLY, GZMB, and NCR1 (Figure 1c) [14,17].

The expression of genes, such as PTPRC (CD45), ACTB, CD3D, and CD68, in cell clusters is shown using a t-SNE plot, with almost all cells expressing PTPRC (CD45) and ACTB (housekeeping genes) (Figure S1e), indicating that the cells analyzed are all immune cells. Furthermore, the t-SNE plot was used to display the high expression of corresponding marker genes in each group (Figure S1e) and to further confirm the accuracy of this study in data processing, cell group division, and marker gene identification.

The t-SNE plot shows the distribution of and changes in immune cells in different samples before and after paclitaxel treatment (Figure 1d,e). Subsequently, a bar graph was used to measure the proportion of immune cell groups in each sample pre- and post-treatment (Figure 1f). We found significant changes in the immune cells before and after treatment in the same tumor tissues of paired patients, indicating that paclitaxel treatment can cause changes in immune cells but that the changes vary in different patients.

### 2.2. Correlation between Treatment Efficacy and Changes in Proportions of Immune Cells

**Figure 2:**
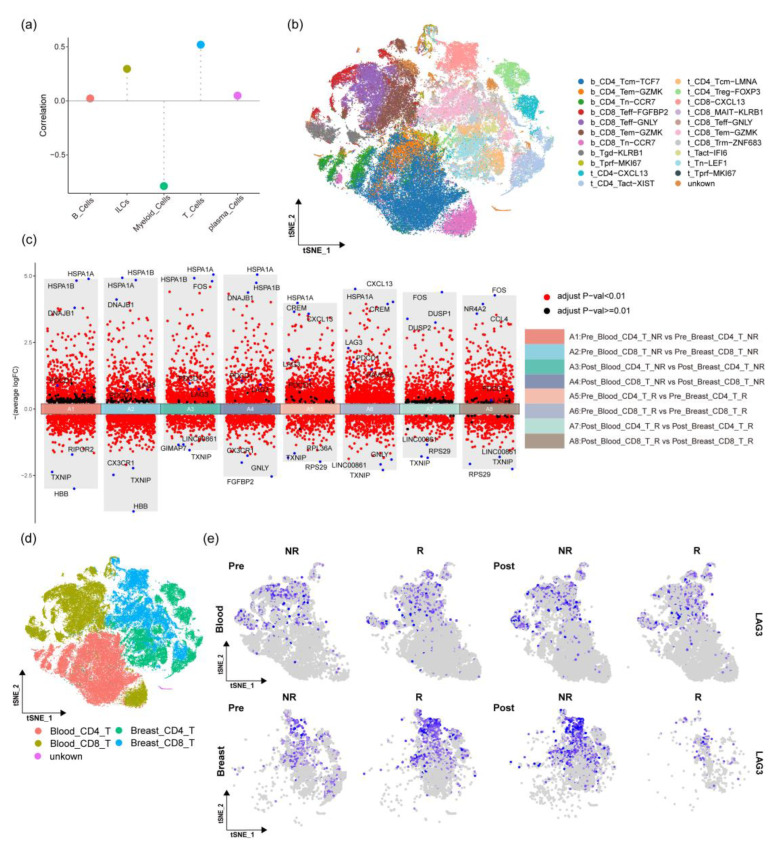
The relationship between changes in immune cells and treatment efficacy, as well as the variations in T cells across different groups. (**a**) Correlation between immune cell changes and relative changes in tumor biopsy lesions. (**b**) Displaying the types and distribution of T cell subgroups through the t-SNE plot. (**c**) Comparing the differences in CD4^+^ and CD8^+^ T cells under different conditions (The blue dots represent the annotated genes, and the red dots represent the differentially expressed genes). (**d**) Distribution of CD4^+^ and CD8^+^ T cells in tumors and blood on the t-SNE plot. (**e**) Expression of LAG3 in tumors, blood, and effective and ineffective treatment conditions, as shown in the t-SNE plot.

In order to determine which types of immune cells play a dominant role in the treatment process, we explored the relationship between the changes in the proportion of immune cells before and after treatment, and treatment outcomes. The relative changes in tumor biopsy lesions before and after treatment with paclitaxel for 8 weeks were analyzed. The changes in the proportion of immune cell populations obtained from the aforementioned analysis before and after treatment were calculated. The correlation between the two was analyzed using r = cor (x, y) (where x represents the difference in the proportion of immune cells and y represents the changes in tumor biopsy lesions before and after treatment). We found that T cells and ILCs (Figure 2a) are the dominant immune cells related to treatment outcomes. Therefore, T cells and ILCs were picked to be analyzed further.

### 2.3. The Changes in T Cells in Different Tissues

We performed unsupervised clustering analysis of T cells and subdivided the cells into subgroups consistent with the original T cell subtypes [14], and the distribution of each subgroup was displayed using t-SNE plots (Figure 2b). The T cells were classified into blood_CD8_T, blood_CD4_T, breast_CD8_T, and breast_CD4_T cells (Figure 2d). Notably, distinct distribution patterns were observed among CD8^+^ and CD4^+^ T cells from different tissue sources, indicating that the genes expressed by CD8^+^ and CD4^+^ T cells vary depending on their tissue of origin.

In order to investigate the differentially expressed genes across various tissues, before and after treatment, and under different treatment efficacies, volcano plot combinations were subsequently used to display the differentially expressed genes in the different groups (Figure 2c). In the ineffective treatment group, A1 represents the difference in CD4^+^ cells from tumor tissues and blood before treatment, A2 represents the difference in CD8^+^ cells before treatment, A3 represents the difference in CD4^+^ cells after treatment, and A4 represents the difference in CD8^+^ cells after treatment. In the effective treatment group, A5 represents the difference in CD4^+^ cells before treatment, A6 represents the difference in CD8^+^ cells before treatment, A7 represents the difference in CD4^+^ cells after treatment, and A8 represents the difference in CD8^+^ cells after treatment. Overall, the top differential genes in groups A1-A4 were roughly the same, while in groups A5-A8, the top differential genes were partially the same. Comparing A1 with A3, we found that the high levels of differential genes were not affected before and after treatment in the non-responsive group, and the same phenomenon was observed when comparing A2 with A4. Comparing A5 with A7, we found that the high levels of differential genes changed before and after treatment in the responsive group, and the same phenomenon was observed when comparing A6 with A8.

We further explored the differentially expressed genes. In groups A5, A6, A7, and A8, genes such as CXCL13, HSPA1A, CREM, CCL4, and FOS were highly expressed in CD8^+^ or CD4^+^ T cells in tumor tissues. CXCL13 affects T cell differentiation and is a chemokine for B cells to migrate to the germinal center [18,19]. HSPA1A is upregulated in CD4^+^ and CD8^+^ T cells after treatment with immune checkpoint blockers in various cancers, such as renal cancer, lung cancer, and basal cell carcinoma [20]. CREM is a transcription factor cAMP response element modulator and is crucial for the proliferation of double-negative T cells in systemic lupus erythematosus [21,22]. CCL4 is closely related to CD8^+^ T cell infiltration [23]. This indicates that in the effective treatment group, the expression of immune-related genes of CD4^+^ and CD8^+^ cells increased after treatment and the affected genes were different in tumor tissues and blood. This indicates that these differential genes are related to the regulatory role of T cells, the existence of differential genes in tumor tissues, and different treatment effects.

In addition, it was found that the expression of immune checkpoint genes (ICGs) varied under different conditions. The specific expression of LAG3, PDCD1, and HAVCR2 was further demonstrated using t-SNE pre- and post-treatment and in the presence or absence of treatment effects. Overall, LAG3 expression was higher in the pre-breast-R and post-breast-NR groups than in other groups (Figure 2e), with no difference in expression in the pre-blood-NR, pre-blood-R, post-blood-NR, and post-blood-R groups. The expression of PDCD1 and HAVCR2 was also displayed in the t-SNE plot (Figure S1g,h). It was found that PDCD1 and HAVCR2 were similarly highly expressed in the pre-breast-R and post-breast-NR groups compared to the other groups. Additionally, there was no difference in expression among the pre-blood-NR, pre-blood-R, post-blood-NR, and post-blood-R groups, consistent with the expression pattern of LAG3 across the groups. This indicates that ICGs were highly expressed in the effective treatment group before treatment. In the ineffective treatment group, ICG expression in tumor T cells increased after treatment. However, ICG expression in blood T cells was not affected by treatment.

### 2.4. The Changes in ILCs before and after Paclitaxel Treatment

**Figure 3:**
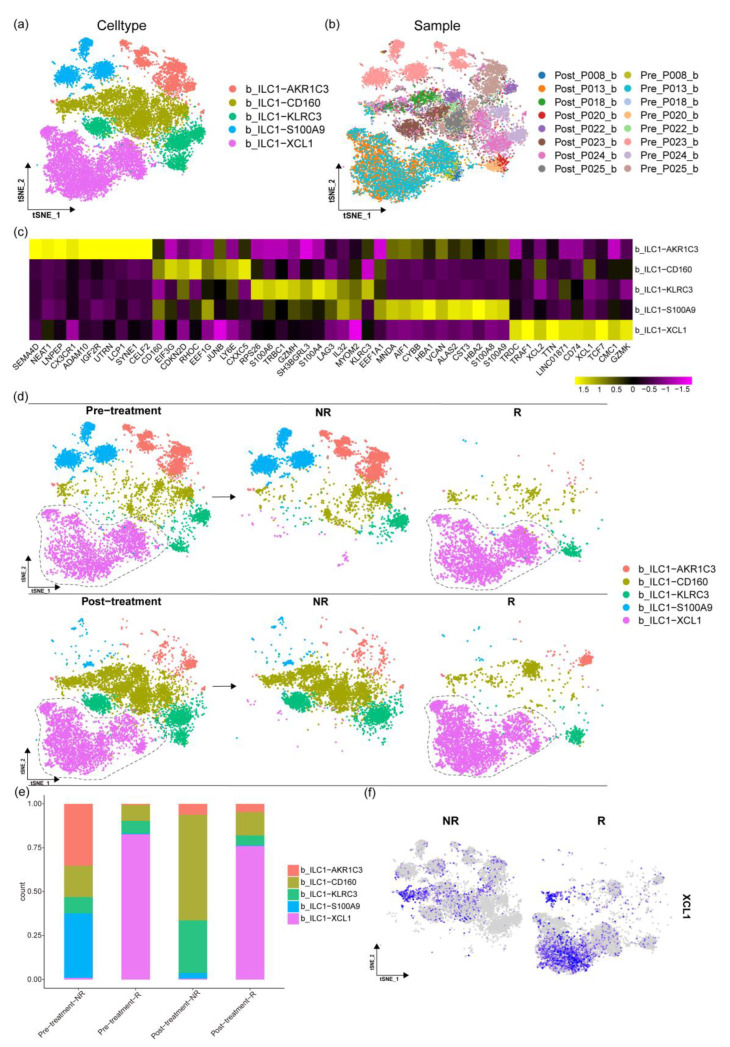
Analysis of ILC subgroups. (**a**,**b**) Displaying the distribution and types of ILC subgroups and the distribution of different samples through the t-SNE plot. (**c**) Heatmap showing the expression of marker genes in ILC subgroups. (**d**) Displaying the changes in ILC subgroups before and after treatment and in different treatment effects through the t-SNE plot. (**e**) Statistics on the proportion of ILC subgroups before and after treatment and in different treatment effects. (**f**) Plotting the expression of the XCL1 gene in different treatment effect groups.

Due to the low number of ILCs in the tumor tissues, only ILCs in the blood were analyzed (Figure 1f). Based on the marker genes tested, the ILC subgroups were divided into b_ILC1-AKR1C3, b_ILC1-CD160, b_ILC1-KLRC3, b_ILC1-S100A9, and b_ILC1-XCL1, and the distribution and types of cell subgroups were displayed using a t-SNE plot (Figure 3a,b). At the same time, a heatmap was used to display the marker genes in each subgroup (Figure 3c).

Enrichment analysis was further performed on the highly expressed genes in each subgroup, and a heatmap was drawn based on the GSEA results. In b_ILC1-AKR1C3, genes such as SEMA4D, NEAT1, and CX3CR1 were highly expressed, which are related to the expression of tumor necrosis factor superfamily cytokines, the transforming growth factor beta receptor signaling pathway, and peptidyl tyrosine modification and type I interferon production. In b_ILC1-CD160, genes such as CD160, EEF1G, and LY6E were highly expressed, participating in pathways such as the long-chain fatty acid metabolic process, the olefinic compound metabolic process, regulation of the steroid biosynthetic process, and positive regulation of macrophage activation. In b_ILC1-S100A9, genes such as S100A9, S100A8, and HBA2 were highly expressed, which are related to the cellular response to interferon beta, the intrinsic apoptotic signaling pathway, and the tumor-necrosis-factor-mediated signaling pathway. In b_ILC1-KLRC3, genes such as KLRC3, TRBC1, and SH3BGRL3 were highly expressed, which are related to regulation of the unsaturated fatty acid biosynthetic process, regulation of the fatty acid metabolic process, and positive regulation of the small-molecule metabolic process. In b_ILC1-XCL1, which is similar to the ILC2a cells found in liver cancer [24], genes such as XCL1, TCF7, and CD74 were highly expressed. The b_ILC1-XCL1 group is supposed to participate in processes such as the inflammatory response, regulation of T cell activation, positive regulation of the immune response, and B cell activation [25].

Subsequent comparison of subpopulation changes under different conditions revealed that the b_ILC1-XCL1 subpopulation was only present in the pre-treatment-R group, with little being found in the pre-treatment-NR group. After chemotherapy, the b_ILC1-XCL1 subpopulation was also only found in the post-treatment-R group (Figure 3d). This is consistent with the results shown in the bar graph depicting the proportions of each subpopulation (Figure 3e). This suggests a correlation between b_ILC1-XCL1 and the responsive treatment.

Upon investigating the marker genes in b_ILC1-XCL1, it was found that the XCL1 gene is highly expressed in the b_ILC1-XCL1 subpopulation (Figure 3f). To explore the relationship between the XCL1 gene and patients, a survival curve of patients with the XCL1 gene was plotted using TNBC data from TCGA [26]. Patients with a high expression of XCL1 had a higher survival rate. This demonstrates the correlation between the b_ILC1-XCL1 subpopulation, marker genes, and patient survival rates.

### 2.5. Cell Trajectory Analysis of ILC Subgroups

**Figure 4:**
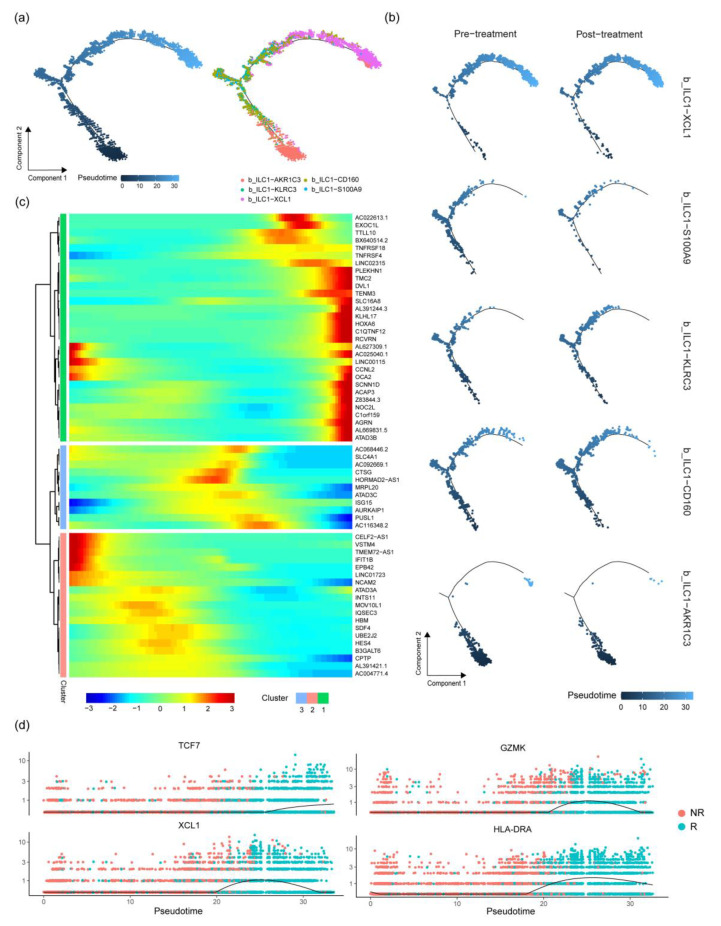
Cell trajectory analysis in ILC subgroups. (**a**) Changes in the cell trajectory of ILC subgroups, represented by varying shades of color to indicate pseudo-time changes. (**b**) Changes in the cell trajectory of various ILC subgroups in different groups before and after treatment. (**c**) A heatmap showing gene expression in ILC subgroups as cell trajectory changes. (**d**) Expression of GAMK, HLA-DRA, TCF7, and XCL1 genes in the context of treatment effectiveness or ineffectiveness and changes in cell trajectory.

**Figure 5:**
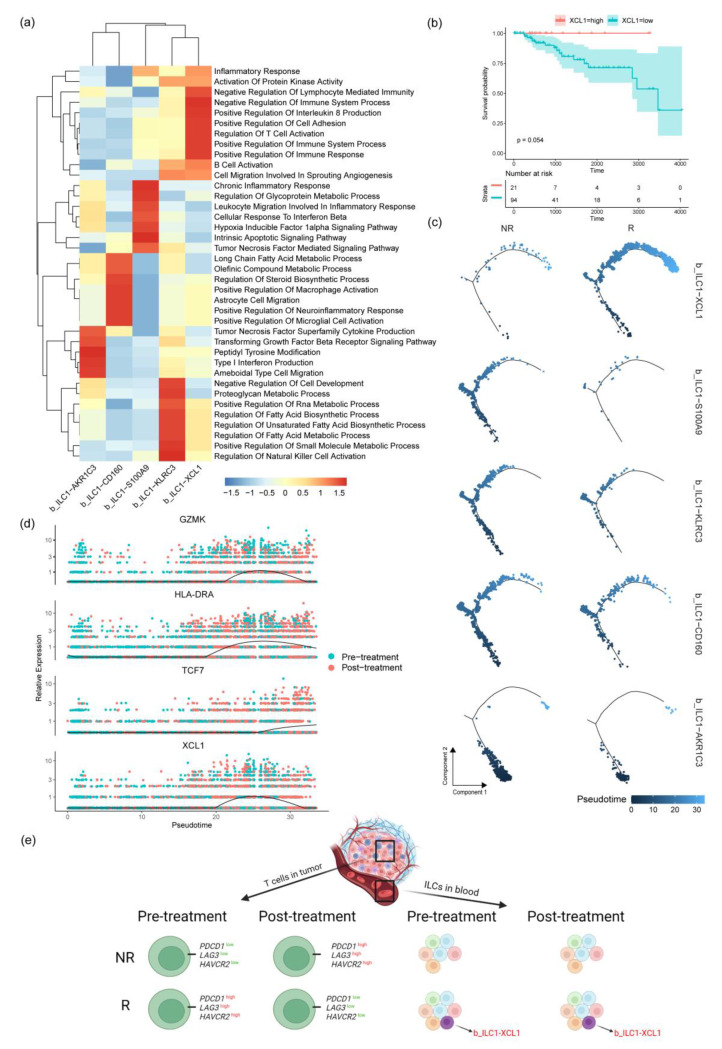
Gene enrichment analysis results and cell trajectory analysis in ILC subgroups. (**a**) A heatmap showing the enrichment results of TOP genes in ILC subgroups using GSAV. (**b**) Survival curve of the XCL1 gene drawn using the Kaplan–Meier survival method. (**c**) Distribution of each ILC subgroup in the cell trajectory in the effective and ineffective treatment groups. (**d**) Expression of GAMK, HLA-DRA, TCF7, and XCL1 genes before and after treatment and changes in the cell trajectory. (**e**) TNBC immune microenvironmental changes in ICGs of T cells and the subgroup of ILCs.

To analyze the differentiation trajectory of ILCs, Monocle 2 was used for cell trajectory analysis [27]. The cell differentiation trajectory began with the b_ILC1-AKR1C3 subgroup, followed by b_ILC1-KLRC3 and b_ILC1-S100A9, while b_ILC1-CD160 was located in the middle of the trajectory, with the trajectory ending with the b_ILC1-XCL1 subgroup (Figure 4a). By comparing the differences in the differentiation process before and after treatment, it was found that b_ILC1-AKR1C3 at the front end of the cell trajectory was higher before treatment than after and that b_ILC1-XCL1 at the end of the cell differentiation trajectory was higher after treatment than before (Figure 4b). At the same time, when comparing the differences in trajectory differentiation between effective and ineffective treatment groups, b_ILC1-XCL1 significantly increased in the effective treatment group (Figure 5c).

The study of gene changes during the process of cell trajectory differentiation divided the genes involved in the differentiation process into three parts. The first part involved genes whose expression gradually increased with cell trajectory differentiation. These genes are associated with cytokine–cytokine receptor interaction, tumor-necrosis-factor-activated receptor activity, receptor clustering, and so on (Table 1). The second part involved genes whose expression first increased and then decreased with cell trajectory differentiation. These genes are related to protein binding and mitochondrial functions. The third part involved genes whose expression gradually decreased following cell trajectory analysis. These genes are related to the integral component of the membrane, plasma membrane, ATP binding, etc. (Figure 4c). Subsequently, changes in the genes related to immune cell toxicity and migration, such as XCL1, GZMK, and TCF7, were found during the trajectory differentiation process (Figure 4d and Figure 5d). This indicates that overall, the changes in cell trajectory are associated with a gradual increase in the genes related to cell toxicity and migration.

## 3. Discussion

TNBC poses a significant therapeutic challenge due to its inherent heterogeneity and the absence of clear molecular targets. Currently, chemotherapy remains the primary treatment approach for TNBC [28]. However, recurrent and metastatic TNBC tends to progress rapidly and often exhibits strong resistance to chemotherapy [29]. Given that chemotherapy affects both immune cells within tumor tissues and the circulating system, information regarding changes in immune cells following chemotherapy is particularly useful for interpreting therapy responses.

In this study, we reanalyzed single-cell data from TNBC before and after paclitaxel treatment. Preliminary cell population divisions were made based on marker genes in cell populations [17]. After confirming the accuracy of this single-cell data analysis through various quality control indicators and the expression of marker genes in each cell population (Figure S1a–d), we established clinical information about changes in immune cells before and after treatment (Figure 2a). As a matter of fact, T cells and ILCs had the highest correlation with the relative changes in tumor biopsy lesions before and after treatment. These data are consistent with previous studies on TNBC that have indicated that γδT cells influence TNBC progression [30], and autophagy defects play a pivotal role in TNBC’s evasion of T cell immune attacks [31] and underscore the importance of T cells in TNBC development and therapy.

In terms of T cell subgroups, we compared the changes in CD4^+^ and CD8^+^ T cells before and after treatment. We found that the highly differential genes were essentially consistent in the ineffective treatment group. However, the highly differential genes changed in the effective treatment group. In the effective treatment group, the expression of inflammation, cytokines, and Toll-receptor-related genes (such as FOS, CCL4, and NR4A2) increased in the CD8^+^ T cells in tumor tissues after treatment. This enhanced the anti-tumor ability of immune cells in the tumor tissues. In contrast, we did not see any change in blood immune cells before and after treatment.

Additionally, immune checkpoint genes (ICGs) play critical roles in maintaining immune homeostasis and preventing autoimmunity. They are expressed on cells of both the adaptive immune system, particularly T cells, and the innate immune system. For instance, LAG3 expression is inversely related to the immunoregulatory function of antigen-specific T cells. Blocking LAG3 can boost the anti-tumor effects of CD8^+^ T cells in an antigen-specific manner [32]. We observed differences in the expression of ICGs. LAG3, PDCD1, and HAVCR2 were expressed at higher levels in the pre-breast_R and post-breast_NR groups compared to other groups. This suggests that high expression in the effective treatment group of tumor tissues before treatment (pre-breast-R) may yield positive treatment outcomes for patients. Conversely, high expression in the ineffective treatment group of tumor tissues after treatment (post-breast-NR) may be due to increased T cell exhaustion induced by treatment, since the expression of exhaustion markers in the later stages of treatment indicates adverse clinical outcomes [33]. LAG3, PDCD1, and HAVCR2 genes exhibit the same expression pattern (Figure 2e, Figure 5e and Figure S1g,h). Concurrently, the LAG-3/PD-1 dual-antibody EMB-02 treatment plan for melanoma has been approved [34], suggesting that the treatment of TNBC through dual-antibody combined chemotherapy warrants further investigation. However, the reason why high ICG expression in the early stages of treatment results in better effects after chemotherapy still requires further investigation. In contrast, we did not see the differential expression of LAG3, PDCD1, and HAVCR2 genes in the blood of any patient groups, suggesting differences between the tumor and blood circulation immune microenvironments.

ILCs can secrete inflammatory mediators similar to T lymphocytes, playing a regulatory or enhancing role in immune responses in cancer [35]. In this study, the ILC subgroups were determined using specific gene markers, and enrichment analysis was conducted on TOP genes to decipher the function of each ILC subgroup (Figure 5a). We found that the b_ILC1-XCL1 subgroup has a high proportion in the pre- and post-treatment groups of responsive patients, suggesting a correlation between the b_ILC1-XCL1 subgroup and treatment efficacy. The highly expressed genes in b_ILC1-XCL1 are involved in inflammatory responses, positive regulation of immune responses, and activation of B cells. We also investigated the characteristic gene XCL1 in the b_ILC1-XCL1 subgroup. XCL1 is a chemokine that can induce leukocyte migration and activation when secreted by various immune cells [36], and XCL1 expression correlates with CD8-positive T cell infiltration [37]. Survival analysis reveals that patients with high XCL1 expression have a higher survival rate (Figure 5b). Moreover, the b_ILC1-XCL1 subgroup gradually increases following cell trajectory differentiation after treatment (Figure 5e). These data suggest that the b_ILC1-XCL1 subgroup and the marker gene XCL1 may serve as preliminary diagnostic markers for treatment efficacy in TNBC patients before treatment.

In conclusion, the reanalysis of TNBC data revealed the functional differences between CD4^+^ and CD8^+^ T cells, with differential gene expression in tumor tissues before and after paclitaxel treatment. The immune microenvironment in patient blood is largely unaffected. The expression of ICGs is correlated with different treatment effects. High ICG expression in the pre-treatment effective group correlates with the increase in ICG expression in tumor tissues caused by paclitaxel chemotherapy, providing a theoretical basis for combined treatment with two or more antibodies and chemotherapy. Finally, the b_ILC1-XCL1 subgroup and the marker gene XCL1 in the ILC subgroup can serve as gene diagnostic markers for the effectiveness of paclitaxel chemotherapy.

## 4. Materials and Methods

### 4.1. Single-Cell Data Analysis

Single-cell sequencing data of triple-negative breast cancer were obtained from the GEO database GSE169246. A total of 8 TNBC patients were used in this analysis, with relevant clinical characteristics provided in Table S1. The data matrix was read using the Seurat package (v4.2.0) in R for unsupervised clustering analysis. Following the Seurat tutorial, the data matrix was created, and cells with >10% mitochondrial genes, >6000 genes, or <200 genes were excluded. The “NormalizeData” function was used to normalize gene expression, and the “FindVariableFeatures” function was used to obtain the 2000 most variable genes for principal component analysis. t-SNE was used to visualize the distribution of cell clusters. The “FindAllMarker” function was used to identify the marker genes of the cell clusters, and feature plots and scatter plots were generated.

### 4.2. Correlation Analyses of Treatment Effects and Immune Cell Proportion Changes

The changes in immune cells before and after treatment in multiple paired samples were calculated, as well as the relative changes in tumor biopsy lesions. The correlation between the two was calculated using the cor function (v4.1.2) in R, r = cor(x,y) (where x represents the difference in the proportion of immune cells before and after treatment and y represents the change in tumor biopsy lesions before and after treatment). The ggplot2 package was used to plot a dot plot of each immune cell and its correlation.

### 4.3. Cell Developmental Trajectory

The developmental trajectory of ILCs was analyzed using Monocle 2 (v2.22.0), following the guidelines provided in the Monocle 2 tutorial. The “differentialGeneTest” function was used to identify genes that were differentially expressed between cell clusters. Following this, genes with a q-value of less than 0.01 were selected for further trajectory analysis.

### 4.4. Pathway Analysis and Functional Annotation

Gene Ontology enrichment analysis and single-sample Gene Set Enrichment Analysis (ssGSEA) were used for functional analysis. The gene signature scores of the samples were evaluated using the GSVA package (v1.42.0) in R.

### 4.5. Survival Analysis

The Kaplan–Meier survival method was used to analyze the prognosis between groups using the “survival” package (v3.213) in R. The log-rank test was used to calculate the differences in survival curves.

### 4.6. Code Availability

The analysis scripts for this project are available at https://github.com/zh1221/TNBC_single (accessed on 25 July 2023). This GitHub repository contains the R code used for the data analysis.

## Figures and Tables

**Table 1:** Results of three-part gene enrichment analyses with changes in cell trajectory.

	Term	ID	Input Number	Background Number	*p*-Value	Corrected *p*-Value	Input
Part I	Cytokine–cytokine receptor interaction	hsa04060	2	294	1.29 × 10^−2^	4.94 × 10^−2^	TNFRSF4|TNFRSF18
	Tumor-necrosis-factor-activated receptor activity	GO:0005031	2	10	2.16 × 10^−5^	5.63 × 10^−3^	TNFRSF4|TNFRSF18
	Receptor clustering	GO:0043113	2	26	1.23 × 10^−4^	1.58 × 10^−2^	AGRN|DVL1
	Neuromuscular junction development	GO:0007528	2	32	1.82 × 10^−4^	1.58 × 10^−2^	AGRN|DVL1
Part II	Protein binding	GO:0005515	5	11,779	2.89 × 10^−2^	4.04 × 10^−2^	AURKAIP1|SLC4A1|ISG15|MRPL20|CTSG
	Mitochondrion	GO:0005739	3	1258	1.05 × 10^−3^	1.20 × 10^−2^	AURKAIP1|ATAD3C|MRPL20
	Mitochondrial inner membrane	GO:0005743	2	385	1.97 × 10^−3^	1.30 × 10^−2^	AURKAIP1|MRPL20
	Mitochondrial translational elongation	GO:0070125	2	86	1.04 × 10^−4^	5.80 × 10^−3^	AURKAIP1|MRPL20
Part III	Integral component of membrane	GO:0016021	5	3643	9.36 × 10^−3^	2.89 × 10^−2^	ATAD3A|VSTM4|B3GALT6|NCAM2|UBE2J2
	Plasma membrane	GO:0005886	5	4619	2.46 × 10^−2^	5.12 × 10^−2^	VSTM4|SDF4|EPB42|NCAM2|CPTP
	ATP binding	GO:0005524	4	1463	1.90 × 10^−3^	2.54 × 10^−2^	MOV10L1|EPB42|ATAD3A|UBE2J2
	Membrane	GO:0016020	3	2075	4.18 × 10^−2^	6.76 × 10^−2^	CPTP|B3GALT6|SDF4

## Data Availability

The data and analysis methods used in this study can be obtained from the corresponding author upon request.

## Supplementary Materials

The supporting information can be downloaded at https://www.mdpi.com/article/10.3390/ijms241814188/s1.
